# Parent-reported autism spectrum disorder (ASD) symptomatology of children with ASD during the COVID-19 pandemic: a 1.5-year longitudinal study

**DOI:** 10.1080/20473869.2024.2418166

**Published:** 2024-10-30

**Authors:** Linde W. E. Hooijman, Ruth Van der Hallen, Anneke Louwerse, Kirsten Visser, Dennis Bastiaansen, Wietske Ester, Elisabeth H. M. van Rijen, Rotterdam Autism Consortium (R.A.C.), Femke L. Truijens, Linda P. Dekker

**Affiliations:** a Department of Psychology, Education & Child Studies, Erasmus University Rotterdam, Rotterdam, The Netherlands; b Rotterdam Autism Consortium (R.A.C.), Rotterdam, The Netherlands; c Department of Child and Adolescent Psychiatry/Psychology, Erasmus MC-Sophia, Rotterdam, The Netherlands; d Parnassia Psychiatric Institute, Youz Child-Adolescent Psychiatry, SARR Expert Centre for Autism, Rotterdam, The Netherlands; e Yulius Autism, Yulius, Mental Health Organization, Barendrecht, The Netherlands; f Child- and Adolescent Psychiatry, Curium-LUMC, Oegstgeest, The Netherlands

**Keywords:** ASD symptomatology, COVID-19, pandemic, SRS, life-event, coping, well-being, family

## Abstract

**Objectives:**

The COVID-19 pandemic and the associative preventive measures have had profound societal impacts. Children and adolescents with a pre-existing diagnosis of autism spectrum disorder (ASD) were considered particularly vulnerable.

**Methods:**

This longitudinal study examined parent-reported ASD symptomatology of children with pre-existing ASD diagnoses during the COVID-19 pandemic. The study involved 39 children and adolescents with ASD (5-19 years; 66% males) and utilized multiple regression analyses to assess ASD symptomatology (SRS-2) across three time points: pre-COVID-19 pandemic (T0), one year into the pandemic (T1; during the second national lockdown) and one and a half years into the pandemic (T2; during a period of less restrictive measures). Additionally, the study examined the potential moderating effects of Age, Sex, and full-scale intelligence quotient (FSIQ) on ASD symptomatology.

**Results:**

The findings indicate relative stability of ASD symptomatology over time, irrespective of age, sex, or FSIQ. Contrary to previous research, no significant exacerbation or alleviation of ASD symptomatology during the COVID-19 pandemic was observed.

**Conclusions:**

This study highlights the robustness of ASD symptomology, even in the face of significant external stressors such as the COVID-19 pandemic. The implications of these findings for further research and clinical practice in the field of ASD are discussed.

## Introduction

On March 11^th^, 2020, the COVID-19 outbreak was declared a pandemic, defined as an event ‘… occurring worldwide, or over a very wide area, crossing international boundaries and usually affecting a large number of people’ (Last 2001; WHO 2020). To prevent the spread of the coronavirus, governments worldwide implemented strict restrictions based on infection rates and the burden on their healthcare systems. Examples of these measures include unprecedented travel restrictions, mandatory social distancing, and the use of personal protective equipment such as facemasks in public areas (Nicola et al. 2020). While effective in containing the virus, these measures—particularly those related to social distancing and movement restrictions—caused substantial collateral damage to society, the economy, the environment, and the humanitarian response system (Haug et al. 2020).

Given its magnitude and duration, the COVID-19 pandemic can be regarded as a significant life event that altered daily life for virtually everyone (Bridgland et al. 2021; Cooke et al. 2020). Previous research has investigated the physical (e.g. Boutin et al. 2021) and psychological effects (e.g. Fisher et al. 2020; Kendrick and Isaac 2020; Kim and Laurence 2020) of the pandemic and the associated restrictions, revealing notable changes in how individuals of all ages functioned, from children to the elderly. Nevertheless, differences in how various subgroups experienced the pandemic are to be expected (Ravens-Sieberer et al. 2022; Shen et al. 2020; Tang et al. 2021). In fact, a narrative review of articles on mental health in children and adolescents suggests that vulnerability factors, such as developmental age and pre-existing mental health conditions, may play a significant role in shaping these experiences (Cost et al. 2022; Singh et al. 2020). Children and adolescents with a pre-existing diagnosis of autism spectrum disorder (ASD) have been suggested to be particularly vulnerable to the challenges posed by the COVID-19 pandemic (Cost et al. 2022; Dogan, Sahin, and Ergenekon 2024; Kerns, Newschaffer, and Berkowitz 2015; Milea-Milea, Fernández-Pérez, and Toledano-González 2024). This heightened vulnerability is hypothesized partly due to a neurobiological predisposition to stress (Spratt et al. 2012), coupled with the many disruptions to one’s routine, health care, and overall limited access to therapy during this period (Bellomo et al. 2020). Consequently, it is reasonable to anticipate changes in ASD symptomatology throughout the pandemic (Dal Pai et al. 2024).

ASD is a complex neurodevelopmental disorder that affects how a person thinks, interacts with others, communicates, and experiences their environment (Neurodevelopmental Disorders 2022). The core characteristics of ASD include difficulties in social communication and interaction, such as difficulties in understanding social cues, maintaining conversations, and forming relationships, and repetitive behaviors and restricted interests, such as repetitive movements, adherence to specific routines, and intense focus on particular subjects or activities. Additionally, many individuals with ASD experience sensory sensitivities, reacting strongly to certain sounds, lights, textures, or other sensory stimuli. The diversity of symptoms and severity levels associated with ASD underscores its classification as a spectrum condition (Neurodevelopmental Disorders 2022). Historically, ASD has been more commonly diagnosed in males than in females (Neurodevelopmental Disorders 2022). However, recent research has highlighted the possibility of a female-typical presentation of ASD, which may result in different expressions of its symptomatology. For instance, some studies suggest that females with ASD encounter more difficulties in social cognition (Di Mento et al. 2024) and social communication (Ros-Demarize et al. 2020), while others report that females tend to exhibit less severe symptoms overall (Stephenson, Norris, and Butter 2023). Moreover, Waizbard-Bartov and Miller (2023) argue that symptom severity in ASD is closely linked to developmental processes and demographic variables such as sex and IQ.

Regardless of these variations in symptomatology, disruptions in routine and unexpected events are generally considered distressing for individuals with ASD, often leading to heightened anxiety and behavioral issues (Fuld 2018). The COVID-19 pandemic and the resulting widespread disruption of daily routines have therefore been identified as significant potential stressors for individuals with ASD (Amorim et al. 2020; Levante et al. 2021; Vasa et al. 2021). Given the crucial role that predictability and routine play in managing ASD, the sudden changes imposed by the pandemic have been suggested to pose considerable challenges for this population. Since the onset of the pandemic in 2020, several studies have documented how the COVID-19 pandemic has affected individuals with ASD. For instance, a retrospective study of 527 Italian parents found that children with pre-existing behavioral problems were twice as likely to experience more intense and frequent behavioral issues during the pandemic (Colizzi et al. 2020). Additionally, some parents reported the emergence of new psychiatric symptoms in their children (Colizzi et al. 2020). Similarly, Renzo et al. (2020) observed a 33% increase in restricted interests and repetitive behaviors (RRB) among 63 Italian children with ASD during the pandemic, which the authors interpreted as an attempt by the children to anchor themselves in familiar routines to counter the many disruptions to their daily routines. Several other studies have since also reported increases in restricted interests, stereotypies, and behavioral problems (Logrieco et al. 2022; Termine et al. 2024). However, the various findings are not entirely consistent with each other. For instance, while Renzo et al. (2020) reported improvements in social communication, both Amaral and de Vries (2020) and Colizzi et al. (2020) found that social skills in children and adolescents declined during the first few months of the pandemic and Siracusano et al. (2021) reported no worsening of repetitive behaviors. In light of these mixed results, researchers have acknowledged the challenges in fully understanding how children and adolescents with ASD have adapted during the pandemic (Hall et al. 2023). These challenges are further complicated by several confounded factors, including pre-existing declines in mental health, restricted access to mental health services during the pandemic, and the lack of pre/post-pandemic measures or longitudinal follow-ups to assess change (Hall et al. 2023).

This innovative study aims to address this challenge by examining parent-reported ASD symptomatology in children and adolescents with ASD during the COVID-19 pandemic using a longitudinal design. Data were collected at three critical time points: pre-COVID-19 pandemic (T0), one year into the pandemic (T1), and one and a half years into the pandemic (T2). The theoretical foundation of our work is built upon three key pieces of evidence. Firstly, ASD is recognized as a neurodevelopmental disorder characterized by relatively stable traits, with core symptoms typically remaining consistent over time (Neurodevelopmental Disorders 2022). This inherent stability is crucial when examining symptomatology in light of external factors, such as a global pandemic. Secondly, existing research indicates that vulnerability factors, including developmental age and pre-existing mental health conditions, significantly influence how individuals with ASD might respond to the challenges posed by the pandemic (Cost et al. 2022; Kerns, Newschaffer, and Berkowitz 2015; Milea-Milea, Fernández-Pérez, and Toledano-González 2024). Consequently, our study focuses on demographic factors and pre-existing ASD symptomatology to better understand how the stressors associated with the pandemic might exacerbate or mitigate ASD symptoms. Finally, we draw upon the Differential Susceptibility Framework (DSF; Belsky, Zhang, and Sayler 2022), which suggests that individuals vary in their sensitivity to both positive and negative environmental influences. This framework provides a basis for understanding why some individuals with ASD may have experienced increased difficulties in social communication and repetitive behaviors during the pandemic, while others may have demonstrated resilience or even improvement in certain areas.

Our primary research questions are: (1) How does ASD symptomatology of children and adolescents with ASD evolve during the COVID-19 pandemic? (2) Are demographic factors such as age, sex, and full-scale intelligence quotient (FSIQ) associated with these changes in ASD symptomatology during the pandemic? Based on our theoretical framework, we hypothesize that: (1) ASD symptomatology will remain relatively stable over time, though certain symptom clusters may intensify or diminish during the pandemic; (2) Demographic factors will be significantly associated with how ASD symptomatology changes during the pandemic. Overall, the COVID-19 pandemic provides a unique context to examine how a major collective life event may influence ASD symptomatology in children and adolescents with ASD. The findings from this study will enhance our understanding of how this population experienced the COVID-19 pandemic and may inform the development of policies and interventions for similar events in the future.

## Method

### Procedure

The present study is part of a broader mixed-method cohort investigation examining changes in the functioning of children and adolescents with ASD and their families during the COVID-19 pandemic (Dekker et al. 2022). The cohort study is a collaborative initiative between Erasmus University Rotterdam and the three main local outpatient mental health care institutions in the area, ensuring a diverse and representative sample of participants from the Rotterdam region. The study protocol was approved by the medical ethical committee of the Erasmus MC (MEC-2020-0720). Screening or Routine Outcome Measurement (ROM) data, including demographic information such as age, sex, and full-scale intelligence quotient (FSIQ), along with the Social Responsiveness Scale (SRS-2), were collected as pre-pandemic baseline data (T0; March 1, 2019, to March 1, 2020) from the three mental health institutions. Follow-up data were collected one year into the COVID-19 pandemic (T1) and one and a half years into the COVID-19 pandemic (T2) using online surveys. These specific time points were selected to best capture how children’s functioning might have varied during different phases of the pandemic, with T1 reflecting a period of stringent restrictions and T2 reflecting a period of eased restrictions where school, work, and social gatherings resumed (albeit with ongoing measures like mask-wearing and limited group sizes). It was hypothesized that the stringent measures during T1 would likely disrupt children’s usual functioning, while T2 would provide insights into how children adapted and whether they showed resilience as the restrictions were lifted (for further detail, refer to https://www.rivm.nl/gedragsonderzoek/tijdlijn-van-coronamaatregelen-2021.

### Participants

A detailed flowchart outlining the inclusion and exclusion criteria of the overall study, along with the response rates, is available elsewhere (Dekker et al. 2022). For this specific study, a flowchart detailing sample sizes and response rates at various stages is provided in Figure 1. At the outset of T1 data collection, email invitations were sent to all parents of children and adolescents diagnosed with ASD at one of our three sites, aged between 4 and 21 years, who had either SRS-2 or CBCL T0 data available (*n* = 213). Follow-up phone calls were made to provide further information and address any questions or concerns. Of the 69 parents who responded to our invitation, 55 (80%) provided informed consent and completed the survey. At T2, all 69 parents were recontacted, and 39 (71%) participated in the survey. Among them, 26 parents (67%) had also completed the T1 survey. At both T1 and T2, participants received a gift voucher upon survey completion. The sample at T1 and T2 was comparable in terms of family structure, level of education for the parents (see Table 1), age, sex, FSIQ, nationality (see Table 2).

**Figure 1.**
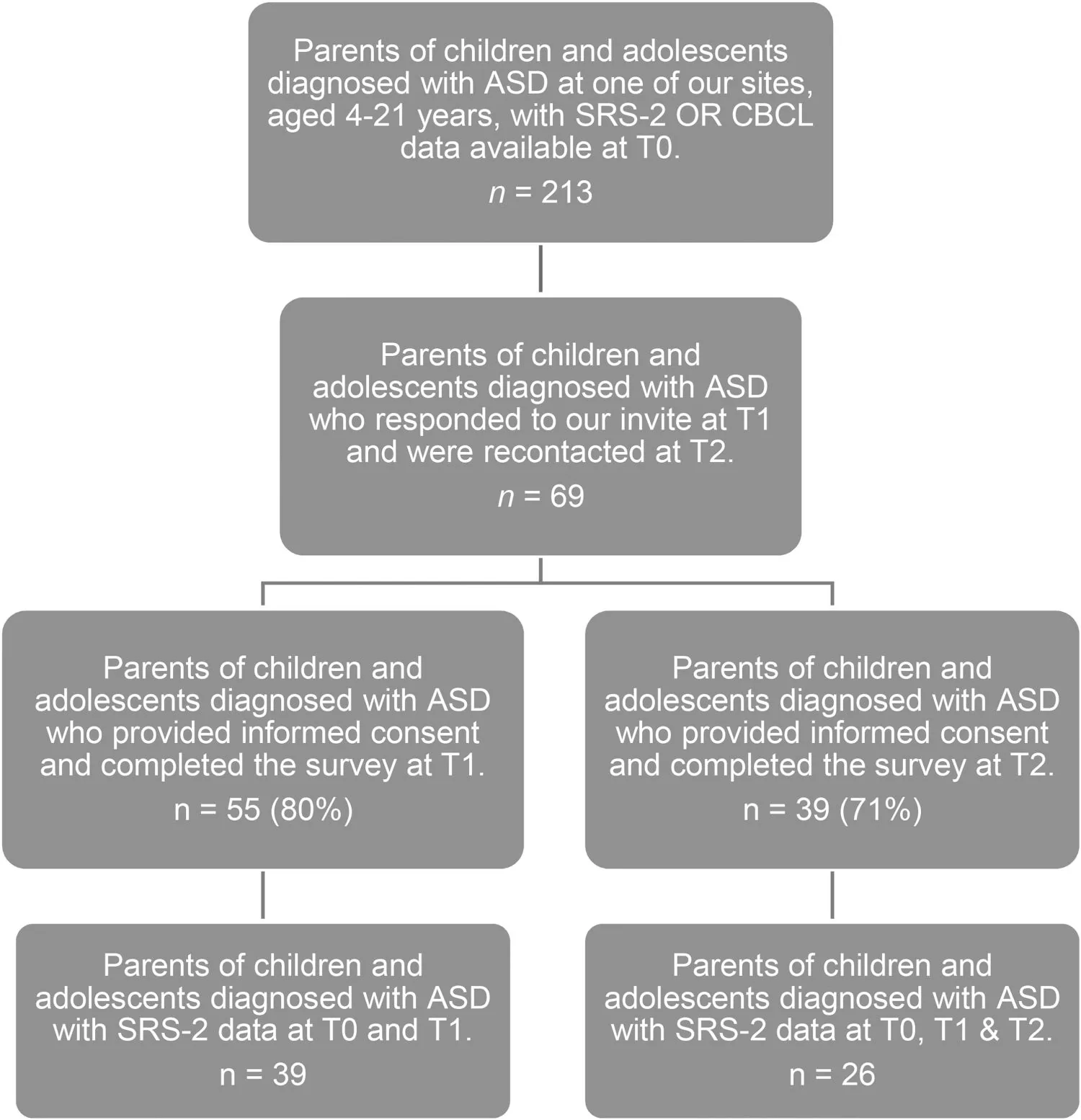
Flowchart of the sample size and response rate at the various stages of the study.

**Table 1.** Demographic characteristics statistics for the sample at T1 versus T2.

	T1 (*n* = 39; 26 boys)	T2 (*n* = 26; 17 boys)
Nationality		
Dutch	78.1%	82.69%
Surinamese	3.85%	1.92%
Turkish	1.28%	1.92%
Cape Verdean	1.28%	0.00%
Unknown	10.26%	7.69%
Family structure		
Two-parent	33.33%	25.64%
Divorced	20.51%	15.38%
Single parent	10.26%	2.56%
Unknown	35.90%	23.08%
Parent educational levels		
Primary education	3.85%	3.85%
Secondary education	38.46%	38.46%
Bachelor’s degree	10.26%	11.54%
Master’s degree	17.95%	21.15%
Unknown	29.49%	25.00%

**Table 2.** Descriptive statistics for the T0-T1 and T0-T1-T2 sample.

*n* = 39; 26 boys	T0			T1		
	M (SD)	Range	T	M (SD)	Range	T
Age (in months)	148.51 (45.73)	70-234				
FSIQ	89.80 (27.59)	27-134				
SRS-2	92.77 (31.28)	30-153	77.46	96.18 (32.93)	21-158	78.79
SCI	76.31 (24.39)	26-122	75.92	78.85 (25.94)	17-126	77.13
RRB	16.46 (8.06)	4-35	79.67	17.33 (8.28)	2-33	81.77

*n* = 26; 17 boys	T0			T1			T2		
	M (SD)	Range	T	M (SD)	Range	T	M (SD)	Range	T
Age (in months)	153.19 (47.93)	83-234							
FSIQ	88.14 (28.03)	27-131							
SRS-2	97.54 (33.49)	30-153	77.46	100.77 (34.32)	29-158	78.79	94.50 (33.50)	17-152	78.08
SCI	79.19 (25.91)	26-122	75.92	81.69 (26.75)	27-126	77.13	76.31 (26.45)	14-123	75.81
RRB	18.35 (8.59)	4-35	79.67	19.08 (8.69)	2-33	81.77	18.19 (8.05)	3-30	83.96

*Note*. SRS-2: Social Responsiveness Scale 2 total score; SRS-SCI: Social Communication and Interaction subscale; SRS-RRB: Restricted Interests and Repetitive Behavior subscale, mean T-scores of 76 and higher indicate severe deficiencies; FSIQ: Full-Scale Intelligence Quotient, FSIQ at T1, *n* = 35, FSIQ at T2, *n* = 22.

### Material

#### Demographics

Sociodemographic characteristics collected at T0 include age, sex, and FSIQ. FSIQ data were retrieved from the electronic patient records, with the most recent intelligence test results used as baseline indicators of intellectual functioning. Intellectual abilities were estimated using various standardized assessments, including the abbreviated version of the Wechsler Preschool and Primary Scale of Intelligence (WPPSI-III-NL; Wechsler, Hendriksen, and Hurks 2011), the Wechsler Non-Verbal (WNV-NL; Wechsler and Naglieri 2008), the Snijders-Oomen nonverbal intelligence test (SON-R; Tellegen and Laros 2011), the revised Amsterdam intelligence test for children (RAKIT-2; Rensin et al. 2012), the Wechsler Intelligence Scale for Children (WISC-III-NL Wechsler 2012), and the Wechsler Adult Intelligence Scale (WAIS-IV-NL; Wechsler 2012). In cases where only age-equivalent IQ norms were available, these norms were converted to estimates of FSIQ.

#### ASD symptomatology

ASD symptomatology was assessed using the Dutch version of the Social Responsiveness Scale-2 (SRS-2; Roeyers et al. 2015), a 65-item questionnaire designed to measure ASD symptomatology. Parents or caregivers rated the presence of ASD symptomatology in their child across different contexts and over time. Each item was rated on a 4-point Likert scale ranging from 0, ‘*Not true*’ to 3, ‘*Always true’*. The SRS-2 includes a total score (SRS-2) and two DSM-5 compatible subscales: (1) Social Communication and Interaction (SCI), and (2) Restricted Interests and Repetitive Behavior (RRB). The SRS-2 has demonstrated good sensitivity, reliability, and validity (Bölte, Poustka, and Constantino 2008).

### Statistical analysis

All analyses were performed using SPSS version 27. To explore how parent-reported ASD symptomatology in children and adolescents evolved during the COVID-19 pandemic, a series of multiple regression analyses was conducted. Prior to performing the regression analyses, multicollinearity diagnostics were employed to ensure the independence of predictor variables. Variance Inflation Factors (VIF) and tolerance values were examined for each predictor variable, with VIF values for all analyses found to be around 1, indicating no multicollinearity concerns. The analysis first focused on the association between data collected at T0 and T1, specifically for SRS-2 total scores and both SRS-2 DSM-5 subscale scores, while considering the role of age, sex, and FSIQ in these relationships. Next, the analysis was extended to include associations between data collected at T0, T1, and T2, again focusing on SRS-2 total scores and the SRS-2 DSM-5 subscale scores, while considering the role of age, sex, and FSIQ in these relationships. For all analyses, a *p* value of *p* < .05 was used to determine statistical significance. Additionally, descriptive analyses, including Pearson correlation, were conducted to analyze the demographic data.

## Results

Descriptive statistics for the SRS-2 total scores and the SRS-2 DSM-5 subscale scores are provided in Table 2. Pearson correlations between the SRS-2, SCI, and RRB scores at different time points were analyzed to understand the stability of ASD symptomatology over time. For the period between T0 and T1, correlations ranged from *r* = 0.539 to *r* = 0.669 (*p* < .01), between T1 and T2 from *r* = 0.494 (*p* < .05) to *r* = 0.604 (*p* < .01), and finally, between T0 and T2 from *r* = 0.636 to *r* = 0.847 (*p* < .01).

A first series of multiple regression analyses was conducted to examine how ASD symptomatology evolved during the COVID-19 pandemic at T1, using SRS-2 data from T0 as the predictor and SRS-2 data from T1 as the outcome variable. The results showed that SRS-2 total scores at T0 significantly predicted SRS-2 total scores at T1, *β* = 0.653, *t*(37) = 4.807, *p* < .001, *R^2^* = .384. Likewise, SCI at T0 significantly predicted SCI at T1, *β* = 0.649, *t*(37) = 4.681, *p* < .001, *R^2^* = 0.372, as did RRB at T0 for RRB at T1, *β* = 0.688, *t*(37) = 5.486, *p* < .001, *R^2^* = .449. Furthermore, the moderate to moderately high *R*^2^ values observed in all three models (*R*^2^’*s* > 0.37) indicate that symptomatology at T0 accounts for a substantial proportion of the variance at T1, underscoring the relative robustness of ASD symptomatology over this period. When age, sex, and FSIQ were added to each regression model, all models remained significant (*p* < .001), but only SRS-2 scores at T0—whether Total, SCI, or RBR—remained significant predictors, while age, sex and FSIQ did not predict symptomatology at T1 for any of the analyses (*p* > .05, *R^2^*’*s* > 0.545). For more detail, see Table 3.

**Table 3.** Multiple Regression Analyses for T0 predicting T1(*n* = 39; 26 boys).

*n* = 39; 26 boys	*B*	*SE b*	*β*	*t*	*df*	*F*	*p*	*R^2^*
Model					4, 30	9.190	**<.001**	.551
Constant	76.417	24.963		3.061			**.005**	
SRS-2	.663	.143	.617	4.640			**<.001**	
Sex	−3.660	7.983	−0.057	−0.458			.650	
Age	−0.112	.083	−0.167	−1.355			.186	
FSIQ	−0.235	.148	−0.211	−1.588			.123	
Model					4, 30	8.973	**<.001**	.545
Constant	58.962	19.195		3.072			**.004**	
SCI	.692	.140	.648	4.947			**<.001**	
Sex	−5.537	6.283	−0.111	−0.881			.385	
Age	−0.084	.065	−0.161	−1.295			.205	
FSIQ	−0.154	.114	−0.177	−1.357			.185	
Model					4, 30	10.394	**<.001**	.581
Constant	16.357	6.336		2.582			**.015**	
RRB	.610	.150	.556	4.080			**<.001**	
Sex	1.728	2.030	.102	.851			.402	
Age	−0.029	.021	−0.161	−1.349			.187	
FSIQ	−0.080	.040	−0.272	−1.990			.056	

*Note*. SRS-2: Social Responsiveness Scale 2 total score; SCI: Social Communication and Interaction subscale; RRB: Restricted Interests and Repetitive Behavior subscale. FSIQ: Full-Scale Intelligence Quotient. Significant *p* value are marked in bold.

A second series of multiple regression analyses was conducted to examine how ASD symptomatology evolved further into the pandemic at T2, using SRS-2 data from both T0 and T1 as predictors and SRS-2 data from T2 as the outcome variable. Note that given the small sample size of 26 at T2, these results should be interpreted with caution. The results showed that a model with SRS-2 total scores at T0 and T1 significantly predicted SRS-2 total scores at T2, *F*(2,23) = 29.451, *p* < .001, *R*^2^ = 0.719, although only T1 SRS-2 total data emerged as a significant predictor of T2 SRS-2 total data (*p* < .001). Similar results were found for the SCI, *F*(2,23) = 28.316, *p* < .001, *R*^2^ = 0.711, and RRB, *F*(2,23) = 34.817, *p* < .001, *R*^2^ = 0.752, subscales, with only T1 data significantly predicting T2 data. Additionally, the high *R*^2^ values across the three models (*R*^2^’*s* > 0.711) indicate that the models using SRS-2 data from T0 and T1 as predictors account for a large proportion of the variance at T2, highlighting the robustness of these relationships over time. Furthermore, when age, sex, and FSIQ were included in each of the models, none of the demographics reached significance, while both T0 and T1 (SRS-2 total/SCI/RRB) data remained significant predictors within the models (*p* < .05, *R*^2^’*s* > 0.793). For more detail, see Table 4.

**Table 4.** Multiple Regression Analyses for T0-T1 predicting T2 (*n* = 26: 17 boys).

	*B*	*SE b*	*β*	*t*	*df*	*F*	*p*	*R^2^*
Model					2, 23	29.451	**<.001**	.719
Constant	5.381	12.921		.416			.681	
SRS-2 T0	.143	.130	.142	1.098			.284	
SRS-2 T1	.746	.127	.765	5.891			**<.001**	
Model					2, 23	28.316	**<.001**	.711
Constant	3.794	10.837		.350			.729	
SCI T0	.126	.132	.124	.953			.351	
SCI T1	.765	.128	.774	5.967			**<.001**	
Model					2, 23	34.817	**<.001**	.752
Constant	1.933	2.184		.885			.385	
RRB T0	.162	.122	.173	1.329			.197	
RRB T1	.696	.121	.751	5.764			**<.001**	
Model					5, 21	12.590	**<.001**	.893
Constant	5.010	29.058		.172			.865	
SRS-2 T0	.404	.150	.382	2.699			**.016**	
SRS-2 T1	.594	.159	.588	3.727			**.002**	
Sex	−4.172	7.499	−0.065	−0.556			.586	
Age	.034	.077	.051	.435			.669	
FSIQ	−0.072	.149	−0.064	−0.484			.635	
Model					5, 21	12.588	**<.001**	.797
Constant	4.571	22.282		.205			.840	
SCI T0	.397	.151	.371	2.622			**.019**	
SCI T1	.623	.159	.599	3.912			**.001**	
Sex	−5.056	5.960	−0.100	−0.848			.409	
Age	.029	.060	.057	.483			.636	
FSIQ	−0.067	.112	−0.075	−0.593			.561	
Model					5, 21	12.270	**<.001**	.793
Constant	.895	6.957		.129			.899	
RRB T0	.340	.149	.341	2.275			**.037**	
RRB T1	.556	.157	.611	3.533			**.003**	
Sex	1.037	1.873	.065	.553			.588	
Age	.005	.019	.030	.254			.802	
FSIQ	−0.012	.041	−0.044	−0.298			.769	

*Note*. SRS-2: Social Responsiveness Scale 2 total score; SCI: Social Communication and Interaction subscale; RRB: Restricted Interests and Repetitive Behavior subscale. FSIQ: Full-Scale Intelligence Quotient. Significant *p* value are marked in bold.

## Discussion and Implications

This study aimed to examine parent-reported ASD symptomatology in children and adolescents with ASD over a period that spanned from pre-pandemic times to one and a half years into the pandemic. Our findings indicate that parents consistently reported mild (*T* = 61 - 75) to severe (*T* > 76) deficits in social responsiveness across both SCI and RRB domains. Notably, these levels of difficulty were observed consistently, even during periods when COVID-19 restrictions were in place. Overall, these findings align with levels of ASD symptomatology typically observed in children and adolescents with ASD under normal circumstances (Roeyers et al. 2015). Additionally, we observed a substantial range in T-scores (*T* = 17 - 152) underscoring the heterogeneity of ASD symptomatology within our ASD sample. By including participants representing the broad spectrum of ASD, our study offers valuable insights into the full range of manifestations exhibited by individuals with ASD during the COVID-19 pandemic, a characteristic that continues to pose significant challenges to clinical practice and scientific research (Masi et al. 2017).

When examining ASD symptomatology over time, our findings suggest that while ASD symptoms remained relatively stable (R2’*s* > 0.70), they were most accurately predicted by the most recent preceding measurement. In other words, ASD symptomatology at T0 best predicted symptomatology at T1, while ASD symptomatology at T1 best predicted outcomes at T2. This pattern was consistent across both SRS-2 total scores and the two SRS-2 DSM-5 subscales, indicating stability across various facets of ASD symptomatology. Interestingly, demographic factors such as age, sex, and FSIQ were not associated with changes in ASD symptomatology over time. This suggests that ASD symptomatology remains relatively stable during prolonged stressful periods, such as the COVID-19 pandemic, irrespective of these demographic factors and that this period cannot be considered a turning point regarding symptom severity (Waizbard-Bartov and Miller 2023). These findings resonate with those of Martínez-González, Moreno-Amador, and Piqueras (2021), who reported relatively stable autistic traits during periods of COVID-19 confinement compared to pre-pandemic times. Overall, these results align with previous assertions that ASD and its associated symptomatology tend to remain relatively stable over time (Benedetto et al. 2021; Neurodevelopmental Disorders 2022; Pierce et al. 2019), highlighting the robust nature of ASD as a phenomenological construct (Kleinman et al. 2008; Matson and Horovitz 2010).

This relative stability in ASD symptomatology over time can be interpreted from different perspectives. On one hand, this stability may reflect the resilience of children and adolescents with ASD, demonstrating their ability to maintain consistent functioning despite the external challenges presented by the COVID-19 pandemic. Such resilience is particularly notable given the significant disruptions caused by the pandemic, including changes in routines and the stress associated with public health measures (Cost et al. 2022; Kerns, Newschaffer, and Berkowitz 2015; Milea-Milea, Fernández-Pérez, and Toledano-González 2024). Although other psycho-emotional domains may have been impacted during this period, as noted by Pellicano et al. (2022), the relative constancy of core ASD symptoms suggests a robust capacity to withstand these external pressures. Another possible explanation for this stability could be rooted in the nature of ASD itself. Children with ASD often experience social isolation and have limited social interactions even outside of a pandemic context (Gómez-Campos et al. 2023; Lois Mosquera et al. 2021). This pre-existing state of reduced social engagement may have buffered them from the stress and disruptions that might have otherwise exacerbated ASD symptoms. Moreover, the stress of isolation may have been less pronounced for these children, as they may have been more familiar with and less disturbed by such circumstances compared to their neurotypical peers.

Additionally, the absence of significant effects related to age, gender, and IQ is particularly noteworthy. This lack of differentiation suggests that these demographic factors do not substantially alter the trajectory of ASD symptomatology during prolonged periods of external stress, such as a global pandemic. One possible reason for this could be that the core features of ASD are deeply ingrained and less susceptible to change based on demographic factors alone, despite previous assertions of their importance (Di Mento et al. 2024; Ros-Demarize et al. 2020; Waizbard-Bartov and Miller 2023). However, it is also important to consider that the relatively small sample size may have limited our ability to detect subtle moderating effects of these variables. Finally, this relative stability raises concerns about the limited adaptability or malleability of ASD symptomatology to external influences. If a significant event like the COVID-19 pandemic did not markedly alter ASD symptoms across different age groups, genders, or IQ levels, it prompts questions about the potential effectiveness of traditional therapeutic interventions or treatment approaches (Bridgland et al. 2021; Cooke et al. 2020). This may indicate that more tailored, intensive or innovative strategies are required to achieve meaningful changes in ASD symptomatology (Pervin, Ahmed, and Hagmayer 2022).

### Strengths and limitations

The current study boasts two significant strengths that enhance the robustness of its findings. Firstly, this study is one of few longitudinal studies to examine changes in ASD symptomatology during the COVID-19 pandemic using pre-pandemic data as a baseline. Unlike studies that rely solely on parental reports of changes observed *during* the pandemic (e.g. Amaral and de Vries 2020; Colizzi et al. 2020; Renzo et al. 2020), this study benefits from having collected data both before and during the pandemic. This unique approach allows for a more accurate comparison of ASD symptomatology over time, offering a clearer understanding of how ASD symptomatology has evolved over time. Secondly, this study included participants from three different outpatient mental health care institutions, which enhances the representativeness of the sample in terms of age, IQ level, and symptomatology. This broad representation not only increases the generalizability of our findings beyond a narrow subset of the autism spectrum (Jensen and Spannagel 2011) but also provides a more comprehensive view of ASD symptomatology over time. By including participants with a wide range of symptom severity, our study captures the diversity of ASD manifestations, offering valuable insights into how children and adolescents with ASD may have responded to the challenges posed by the pandemic.

Despite these strengths, the current study has some limitations that must be taken into consideration. Firstly, the sample size was relatively small, with only 32.4% of the initially invited sample (69 out of 213) agreeing to participate. Moreover, the relatively small sample size may have limited the statistical power needed to detect small to medium moderation effects of sex, age, and IQ on ASD symptomatology. In addition, the participation rate declined over time, with only 39 participants continuing at T1 and only 26 at T2. While speculative, this decrease in participation may have been due to the heightened caregiving responsibilities imposed by the ongoing COVID-19 pandemic. The increased demands on caregivers’ time and attention, coupled with the surge in online activities, might have contributed to participant dropout and a reduced willingness to engage in the study. Alternatively, it is also possible that some parents chose not to participate because the impact of the pandemic may have been less significant, reducing the perceived relevance of the study for them. This dynamic could have potentially introduced biases into the results, consistent with the Differential Susceptibility Framework (Belsky, Zhang, and Sayler 2022), as those who continued participation may differ systematically from those who chose not to, affecting the generalizability of the findings.

Secondly, this study relied exclusively on parent reports. Despite efforts to collect data directly from both parents and children, the response rate from the latter was insufficient for meaningful analysis, as detailed further by Dekker et al. (2022). While parent reports are commonly used and considered valid in assessing children and adolescents with ASD, relying solely on parent reports presents a potential limitation by providing a somewhat one-sided perspective. Especially during the lockdown period, when families spent prolonged periods together at home, heightened parental presence and attention may have influenced parents’ perception of ASD symptomatology of their children (Alhuzimi 2021; Isensee et al. 2022). Additionally, the study did not account for potential adaptations in treatment during the pandemic, such as the use of telemedicine and video consultations, which could have also influenced the observed symptom stability (Ali et al. 2023). This calls for a nuanced consideration – not only of the relative stability of ASD symptoms themselves but also of how families perceive and subsequently report on ASD symptomatology. Recognizing the potential influence of familial dynamics, changes in the home environment, or treatment adaptations on symptom reporting adds complexity to the interpretation of our results and prompts reflection on the interplay between such factors and the traits of ASD.

Thirdly, it is important to note that while our study focused on ASD symptomatology as a measurable outcome, ASD symptomatology does not fully represent the broader mental health or overall well-being of individuals with ASD (Lord et al. 2018; Neurodevelopmental Disorders 2022). Although significant changes in ASD symptoms can offer insights into certain aspects of well-being—given that these symptoms are closely linked to daily functioning and quality of life (Waizbard-Bartov and Miller 2023)—they are not synonymous with well-being or mental health in its entirety. Other dimensions of well-being, such as emotional health, stress levels, and coping mechanisms (Lord et al. 2018), were not directly measured in this study. This limitation underscores the need for future research to incorporate a broader range of mental health indicators to capture the full impact of significant life events like the COVID-19 pandemic on individuals with ASD. A more comprehensive approach, which includes these additional dimensions, could offer a deeper understanding of the challenges faced by this population and inform more targeted interventions. By integrating both ASD symptomatology and broader mental health indicators, future research can provide a more holistic view of the overall well-being of individuals with ASD, particularly during periods of heightened external stress.

### Implications

Altogether, the findings of this study hold important clinical implications for the care of children and adolescents with ASD amidst and following significant stressors such as the COVID-19 pandemic. While ASD symptomatology, as reported by parents, appeared relatively stable throughout the pandemic, it is important to recognize that individuals with ASD may still show vulnerability to challenges such as social isolation, disrupted routines, and associated stress. These challenges may manifest in emotional or behavioral disruptions that are not fully captured by measures of social responsiveness alone (Amirova, CohenMiller, and Sandygulova 2022; Isensee et al. 2022; Smile 2020). Therefore, clinicians should remain mindful of the potential impact of significant life events on the well-being of the ASD population and consider the need for early and/or ongoing interventions (Koegel et al. 2014; Volkmar 2014) to support the long-term well-being of individuals with ASD, extending beyond the scope of social responsiveness.

Direct interventions targeting individuals with ASD have shown significant potential in supporting emotional and behavioral well-being. A review of the literature highlights the effectiveness of cognitive behavioral therapy (CBT) programs in addressing anxiety, particularly for youth with ASD (Perihan et al. 2020; Ung et al. 2015), though the results are less consistent for treating anxiety in adults with ASD (Menezes et al. 2022). Moreover, the use of mentalization-based treatment (MBT) for individuals with ASD has been successful in improving emotional regulation and reducing anxiety among children and adolescents (Menezes et al. 2022). These interventions can serve as an important source of support for individuals with ASD in managing disruptions to their routines and maintaining overall mental health. As such, these types of programs should be considered part of a holistic intervention plan during periods of significant stress.

In addition to direct support for individuals with ASD, it is also important to consider the well-being of parents and caregivers, as they play a vital role in managing the daily challenges faced by their children. Tailored interventions for parents can serve as a buffer during major life events (for a review, see Lichtlé et al. 2020). For instance, the novel 8-week AMOR (Acceptance, Mindfulness, Optimism, Resilience; Schwartzman et al. 2022) method for parent groups has shown promising results for parents of children with ASD. In a randomized trial, parents who participated in the AMOR program reported significant gains in resilience, improved stress management, and reductions in mental health symptoms, underscoring the potential benefits of targeted interventions for parental well-being. These findings highlight the importance of supporting both individuals with ASD and their families, particularly during times of significant disruption.

## Conclusion

This study makes a significant contribution to the literature by examining parent-reported ASD symptomatology of children and adolescents with ASD across time, using a longitudinal approach that spans from pre-pandemic times to one and a half years into the COVID-19 pandemic. By focusing on ASD symptomatology during a period of substantial societal disruption, this research offers valuable insights into the stability of symptoms amidst external challenges. While our findings contribute to a deeper understanding of ASD during these unprecedented times, we acknowledge the necessity of further research with larger samples and more diverse assessment methods, including self-reports, to comprehensively explore the influence of external factors, as well as demographic variables like age, gender, and IQ, on the mental health of individuals on the autism spectrum.

## Data Availability

The data that support the findings of this study are available from the corresponding author upon reasonable request.
